# Association between amebic liver abscess and Human Immunodeficiency Virus infection in Taiwanese subjects

**DOI:** 10.1186/1471-2334-8-48

**Published:** 2008-04-16

**Authors:** Meng-Shuian Hsu, Szu-Min Hsieh, Mao-Yuan Chen, Chien-Ching Hung, Shan-Chwen Chang

**Affiliations:** 1 Far Eastern Memorial Hospital, Taipei, Taiwan; 2 Department of Internal Medicine, Section of Infectious Diseases, National Taiwan University Hospital, Taipei, Taiwan

## Abstract

**Purpose:**

Invasive amebiasis is an emerging parasitic disorder in Taiwan, especially in patients diagnosed with human immunodeficiency virus (HIV) infection. Thirty-three Taiwanese subjects with amebic liver abscess (ALA) were examined and a possible correlation between ALA and HIV infection was investigated.

**Results:**

Among ALA patients, the proportion of HIV-positive individuals increased during the study period. ALA was the first major clinical presentation in 54% of HIV patients with ALA. Overall, 58% (14/24) of HIV-infected patients had a CD4+ count > 200 cells/μL and 82.1% (23/28) had no concurrent opportunistic infection or other evidence of HIV infection. There was no marked difference in clinical characteristics between HIV-positive and HIV-negative ALA patients except the level of leukocytosis.

**Conclusion:**

While the clinical characteristics described herein cannot be used to determine whether ALA patients have HIV infection, routine HIV testing is recommended in patients with ALA, even in the absence of HIV symptoms.

## Background

Amebiasis, a parasitic infection caused by *Entamoeba histolytica*, is transmitted by ingestion of food or water containing the cyst form of the parasite, and results in amebic colitis and the formation of amebic liver abscess (ALA). Amebiasis is one of the most infectious diseases in the world with developing countries, including Central and South America, tropical Asia, and Africa, as areas of highest incidence. In developed countries, individuals at increased risk for amebiasis include immigrants from developing countries, travelers to the tropics, residents of institutions for mentally retarded individuals, homosexual men, and immunodepressed individuals. Patients with advanced human immunodeficiency virus (HIV) represent one of the highest risk groups of invasive amebiasis [1,2]. Luminal agents such as paromomycin, diloxanide furoate, and iodoquinol are typically used for the treatment of amebiasis caused by *E. histolytica *infection [1].

ALA is the most common extraintestinal manifestation of *E. histolytica *infection. *E. histolytica *transmission is associated with the oral-fecal pathway and is facilitated by poor sanitary conditions [3]. Traveling to endemic areas, with the possibility of ingesting contaminated food or water, remains a risk for contracting ALA. In Taiwan, the improved sanitary system has decreased invasive amebic infection to less than one case per year [4-6]. However, the reappearance of ALA after 1995 appears to be closely associated with HIV infection. The proportion of recent cases of ALA with underlying HIV infection increased markedly.

Recently, Hung et al. have noted that invasive amebiasis has become an emerging parasitic disease in HIV-infected patients in Taiwan [4,5]. The characteristics of ALA in Taiwan remain unknown. Therefore, we conducted a retrospective study to identify the characteristics of ALA and the relationship with HIV infection in Taiwan.

## Methods

### Patients and data collection

Patients who were diagnosed with ALA at the National Taiwan University Hospital (a 2500-bed university hospital and largest referral center for HIV/AIDS in Taiwan) were enrolled from March 1, 1990 to February 28, 2006 in this retrospective study. Detailed medical records, including demographic data, disease status, clinical presentation, risk factors of HIV infection, laboratory findings, treatment, and outcome were reviewed. CD4 count, plasma viral load, and concomitant opportunistic infection(s) records were analyzed if HIV infection was diagnosed. The study described herein was approved by the Institutional Review Board and was in accordance with the Declaration of Helsinki.

### Diagnostic criteria

Study patients were considered to have ALA if image studies had demonstrated the presence of intra-hepatic abscesses and were accompanied with histological, serological, or clinical evidence of amebic infection [4-6]. Three typical conditions were classified as ALA. First, patients with histological evidence of erythrophagocytic trophozoites identified in aspirates or biopsy tissue were diagnosed with ALA. Second, patients with a titer over 1:128 in the indirect haemagglutination (IHA) serologic assay were also included in the group of ALA cases. Third, patients without underlying biliary tract disease who responded well to metronidazole monotherapy, but had positive image studies were also considered as ALA cases, even if their serum IHA titer was less than 1:128 (because the IHA titer assay only has an intermediate sensitivity). Patients who responded well to metronidazole monotherapy were defined as those individuals in which fever or other clinical symptoms/signs improved after 3 days of metronidazole monotherapy. In addition, patients who had specimen such as blood or aspiration fluid with fungus or bacteria pathogens that were concomitantly isolated, were excluded from this study to avoid the enrolment of patients with mixed infection. If ALA recurred during the study period, data for the first time diagnosis was collected.

Patients were considered to have concomitant amebic colitis if trophozoites were found in stools or biopsy specimens from colon fibroscopic examination.

### Statistical analysis

Statistical analyses were performed using SPSS 10.0 for Windows (SPSS, Chicago, Illinois). Statistical significance of continuous variables was determined by using a non-parametric test (Mann-Whitney U test) and categorical variables were determined using the Fisher's exact test. All tests were two-tailed and *P *< 0.05 was considered statistically significant. Continuous variables were reported as mean ± standard deviation (SD).

## Results

### Increase in HIV infection in ALA patients

**Figure 1:**
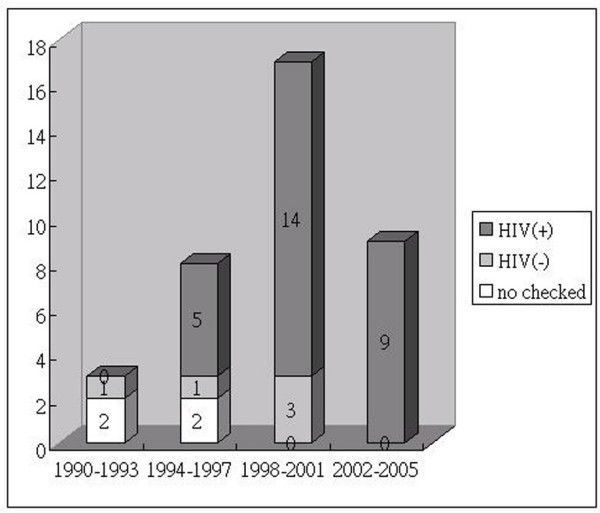
**Cases of ALA at the National Taiwan University Hospital from 1990 to 2005**. White bars represent ALA patients with unknown HIV status; light-gray bars represent HIV-negative ALA patients; dark gray bars represent HIV-positive ALA patients. The proportion of HIV-infected patients among all cases of ALA increased significantly (0% → 62.5% → 82.3% → 100%; *P *= 0.005 by 2 × 4 Chi-square analysis).

During the study period, 37 patients met the diagnostic criteria for ALA. HIV serum test results were obtained in 33 patients. Five (15.2%) patients had negative HIV test results and 28 (84.8%) patients were HIV-positive. Between 1990 and 1994, there was only one case of ALA every year; most patients had an unknown HIV infection status. In 1995, three cases of ALA were recorded, two of which had HIV-coinfection. Since then, the number of ALA cases increased gradually. In 1999, six patients had ALA, with five (82.4%) being coinfected with HIV. After 2002, the rate of ALA patients coinfected with HIV increased to 100% (9/9) (Figure 1). This significant increase prompted us to determine the characteristics of ALA patients with and without HIV infection.

### Demographic characteristics of patients with and without HIV infection

The demographic characteristics of patients with and without HIV infection are listed in Table 1. The mean age was 44.2 ± 10.3 years in HIV-negative patients and 36 ± 10.1 years in HIV-infected patients. No female patients were included in either of the above mentioned groups. The five HIV-negative patients had a history of traveling to or living in endemic areas, including Thailand (n = 1), Burma (n = 2), Pakistan (n = 1), and the Philippines (n = 1). Of the 28 HIV-infected patients, four (14%) had traveled to Thailand within one month of the ALA episode, and 24 (86%) had a history of homosexual behavior. Remarkably, more than half (15/28, 54%) of the HIV-infected ALA patients were found to have HIV infection after they were diagnosed with ALA. Ten (36%) patients developed ALA after HIV had been diagnosed (2 months to 10 years later). The remaining three (10%) patients were found to be HIV-infected 4 months to 3 years after the diagnosis of ALA. Among these three patients, one was diagnosed with HIV infection during the second recurrence of ALA while the other two were diagnosed with HIV infection during other opportunistic infection episodes.

**Table 1:** Characteristics of ALA in patients with or without HIV infection^a^

	**HIV-negative (n = 5)**	**HIV-Infected (n = 28)**	***P *value**
Mean age, years	44.2 ± 10.3 (30–57)	36 ± 10.1 (23–70)	
Male/Female	5/0	28/0	1
IHA titer	512–16384	<8–16,384	
Underlying diseases			
Diabetes mellitus	20% (1)	0 (0)	0.15
HBV hepatitis	20% (1)	32% (9)	1
HCV hepatitis	0 (0)	14% (4)	1
Risk factors			
Endemic traveling	80% (4)	14% (4)	0.08
Clinical manifestation			
Fever	100% (5)	64% (18)	0.29
RUQ pain	40% (2)	57% (16)	0.64
Diarrhea	20% (1)	36% (10)	0.64
Right flank pain	20% (1)	11% (3)	0.5
Concurrent colitis	40% (2)	18% (5)	0.28
Laboratory data			
IHA titer	1:512 ~1:16,384	1:8~1:16,384	
WBC/μL	19,476 ± 9,363	11,982 ± 7,854	0.02
GOT U/L	156 ± 251	52 ± 44	0.94
GPT U/L	106	76 ± 124	0.30
Alkaline phosphatase, U/L	376 ± 324	337 ± 175	0.46
Complications			
Peritonitis	0% (0)	11% (3)	1
Pleural effusions	0% (0)	18% (5)	0.57
Treatment			
Mean Metronidazole use duration (days)	16.8 ± 3.7	14.4 ± 5.6	0.17
Follow-up with idoquinolone	20% (1)	79% (22)	0.01
Outcome			
Recurrence of ALA	20%(1)	7.1% (2)	0.4
Mortality	0% (0)	0% (0)	

^a^Data are presented as mean ± SD, % (n), or range

Of the 28 HIV-infected patients, nine (32%) were hepatitis B virus (HBV) carriers and four (14%) were hepatitis C virus (HCV) carriers. One patient was co-infected with HBV and HDV (hepatitis D virus), and another patient was co-infected with HBV and HCV. Serum Venereal Disease Research Laboratory (VDRL) examination was also positive in 12 (43%) of the HIV-infected patients.

### Significant clinical manifestations

Fever (>38°C) was the most common clinical symptom in ALA patients (100% in the HIV-negative group and 64% in the HIV-infected group, *P *= 0.29). Other symptoms included abdominal pain (40% in the HIV-negative group and 57% in the HIV-infected group, *P *= 0.64) and diarrhea (20% in the HIV-negative group and 36% in the HIV-infected group, *P *= 0.64). Four patients initially presented with right-flank pain and were treated inappropriately for urinary tract infection. The correct diagnosis was obtained later after treatment failed and after image studies were obtained. Some non-specific complaints included cough (n = 6), abdominal fullness (n = 3), anorexia (n = 3), body weight loss (n = 3), and general malaise (n = 3).

Seven patients had amebic colitis diagnosed at the same time (two in the HIV-negative group; five in the HIV-infected group). Five patients were diagnosed with histological findings of amebic trophozoites (four patients via colonoscopy biopsy, one via laparotomy biopsy). Another two patients had trophozoites in their stool specimens. Three patients also had peritonitis complication (one in the HIV-negative group; two in the HIV-positive group). One case of peritonitis was caused by liver abscess rupture and the other case was caused by colitis rupture. None of these patients had empyema, pericarditis, or brain abscess complications.

### Laboratory Examinations

All five HIV-negative ALA patients had IHA titers over 1:128 (from 1:512 to 1:16,384). Of the 28 HIV-infected patients, 24 had IHA titers over 1:128 at the onset of ALA. Four HIV-infected patients had IHA titers remaining below 1:64 during the entire treatment course, but had typically good responses to metronidazole monotherapy.

All patients showed leukocytosis, but the mean white blood cell count in HIV-negative patients was significant higher than that in HIV-infected patients (19,476 ± 9,363/μL [range: 12,930–36,040] versus 11,982 ± 7,854/μL [range: 3,420–38,140], *P *= 0.02). The AST and ALT levels were elevated 3- to 5-fold in HIV-negative patients and 1- to 2-fold in HIV-infected patients, but was not statistically different between the two groups. Both groups had a 2-fold elevation in alkaline phosphate levels (376 ± 324 U/L versus 337 ± 175 U/L). Bilirubin and platelet levels, however, were within normal limits for both groups (i.e., 220 ± 100 K/μL for the platelet level; 0.2–1.2 mg/dL for the bilirubin level).

In the HIV-infected group, the mean CD4 count (data of 24 patients) was 266.95/μL (14 to 798/μL) at the time of ALA diagnosis. Five patients had CD4 counts higher or equal to 350/μL (20%). Nine patients had CD4 counts between 200 and 349/μL (38%). Ten patients (42%) had CD4 counts below 200/μL. Plasma viral loads (data of 18 patients) ranged from undetectable levels (< 400 copies/mL) to > 750,000 RNA copies/mL. Further comparison between patients with IHA titers > 1:128 and patients with IHA titers < 1:64 revealed no significant different in the distribution of CD4 (*P *= 0.39) (Table 2). Five patients developed an opportunistic infection at the same time. One patient had a hairy leukoplakia and his CD4 count was 223/μL. The other four patients had oral candidiasis and their CD4 counts were 14/μL, 84/μL, 229/μL, and 367/μL, respectively.

**Table 2:** CD4 distribution in HIV-infected cases of ALA with available data of CD4+ cell count

	**IHA titer ≥ 128 (n = 20)**	**IHA titer ≤ 64 (n = 4)**
Mean CD4/μL	237.24	415.5
CD4 ≥ 350/μL	3	2
CD4 349 to 201/μL	8	1
CD4 ≤ 200/μL	9	1

### Treatment and outcome

All patients had a good response to metronidazole monotherapy; the mean treatment duration was 14.9 days (range: 7–33 days; 17 ± 3.7 days in HIV-negative patients and 14.4 ± 5.6 days in HIV-infected patients). Eradication therapy with idoquinol was also used in one (20%) HIV-negative patient and 22 (79%) HIV-infected patients (one of whom did not receive idoquinol during the first infection but only when the infection recurred).

The condition of the patients during hospitalization was benign, and all patients were alive at the time of discharge. Follow-up was generally uneventful. ALA recurred in only three patients (one HIV-negative patient and two HIV-positive patients). Of the two HIV-infected patients who had recurrence of ALA, one never received eradication therapy with idoquinol in the first episode. In addition, one patient had recurrent amebic colitis, but no liver abscess despite receiving eradication therapy with idoquinol during the first infection episode.

## Discussion

In the present study, all HIV-infected patients were male. The major risk for acquiring ALA could be attributed to the homosexual behavior (86% in this study). The oral-anal contact renders patients prone to be infected by amebas [4,7,8]. Although studies performed in North America and Europe demonstrated a high rate of ameba carriers (20–30%) among homosexuals, invasive amebic diseases were rare in these reports [2,9-11]. Nevertheless, similarly to the present study, recent studies performed in Asia revealed that invasive amebic diseases occur more often in homosexuals, especially in those homosexual individuals that are coinfected with HIV [2,4,6-8,12]. Whether this is due to the geographic distribution of different zymodemes of ameba, to the homosexual behavior, or to a suppressed host response to *E. histolytica *deserves further study [6]. In addition, some studies suggested that host factors, such as dysregulation of T-cell activity, may also play an important role in HIV-infected patients who are susceptible to invasive amebiasis [2,6].

Previous reports have shown that abdominal pain and fever are two typical clinical symptoms that develop in over 85% patients with ALA [3]. In the present study, we discovered that both fever and abdominal pain occurred less frequently than reported in the literature, especially in HIV-infected ALA patients. Furthermore, we noted that ALA patients coinfected with HIV had a significantly lower white blood cell count than HIV-negative patients. This reduced systemic inflammatory response syndrome (SIRS) may also be attributed to HIV infection. Therefore, the lack of typical ALA symptoms in up to one-third of the patients who participated in this study should alert the physician that further image study may be necessary to rule out ALA in HIV-infected patients with a poor response to current treatment. Most importantly, we showed that it is difficult to determine on the basis of the clinical presentation whether a given patient with ALA exhibits an underlying HIV infection.

In HIV-infected ALA patients, CD4 counts varied greatly from 14 to 798/μL, suggesting that ALA is not caused by an opportunistic infection. In addition, the recurrence of ALA or amebic colitis in HIV patients was rare and was not limited to those who did not receive idoquinol. Therefore, we suggest that the most likely recurrence route was the repeated practice of oral-fecal sex.

Based on our experience, ALA has a benign prognosis in both HIV-negative and HIV-infected patients. A 14-day metronidazole treatment with or without eradication with idoquinol can generally result in a good clinical response. There was no hospital mortality, even in patients with opportunistic infections (n = 5). However, there was a rare occurrence of peritonitis (three patients), which could have been fatal if not adequately treated.

In conclusion, the proportion of HIV infection among ALA patients increased significantly during the study period. No obvious difference in clinical features between HIV-infected and HIV-negative patients could be identified. ALA may be the first major clinical presentation in HIV-infected patients. Most HIV-infected ALA patients had no concurrent opportunistic infection or other evidence of HIV infection. Although this study will need to be expanded in the future including more ALA patients with and without HIV infection, our results strongly suggest that physicians should not rely on the clinical features to determine whether ALA patients are infected with HIV or not. In fact, routine HIV testing is recommended in cases of ALA even in the absence of any clinical features suggesting HIV infection.

## Competing interests

The author(s) declare that they have no competing interests.

## Authors' contributions

HMS, HSM and HCC conceived of the study, participated in the design of the study, coordination, drafted the manuscript, and performed the statistical analysis. All authors participated in management of patients. All authors read and approved the final manuscript.

## Pre-publication history

The pre-publication history for this paper can be accessed here:


